# Determinants of invasive ventilation in infants with acute bronchiolitis: an observational study of pre-hospital and in-hospital treatments

**DOI:** 10.31744/einstein_journal/2025AO1512

**Published:** 2025-10-30

**Authors:** Carlos Henrique Araújo Carvalho, Cristina Ortiz Sobrinho Valete, Carolina Perez Montenegro, Esther Angelica Luiz Ferreira

**Affiliations:** 1 Universidade Federal de São Carlos São Carlos SP Brazil Universidade Federal de São Carlos, São Carlos, SP, Brazil.

**Keywords:** Bronchiolitis, Pulmonary atelectasis, Bronchodilator agents, Epidemiology, Hospitalization, Intubation, Infant, Child

## Abstract

**Objective:**

To analyze the factors associated with invasive ventilation in infants admitted to the hospital with acute bronchiolitis and identify the treatments administered before and during hospitalization.

**Methods:**

This single-center observational retrospective study included 266 infants with acute bronchiolitis who were admitted to the pediatric ward of a university hospital in São Paulo between January 2020 and March 2023. Multivariate logistic regression analysis was used to investigate the factors associated with invasive ventilation, and Prais-Winsten regression was used to analyze the temporal trends of cases. Statistical significance was set at p<0.05.

**Results:**

Of 266 participants, 16 (6%) were intubated. The multivariate analysis revealed that younger age (adjusted odds ratio (AOR)=0.71; 95% confidence interval (95%CI)=0.53–0.96; p=0.02), higher Wood-Downes-Ferres scores at admission (AOR=2.02; 95%CI=1.03–3.95; p=0.03), and the presence of atelectasis on radiography (AOR=26.4; 95%CI=3.85–182.18; p=0.001) were associated with invasive ventilation. Increased bronchodilator use (30.0% *versus* 79.7%, p<0.001) and decreased corticosteroid use (27.8% *versus* 25.5%, p=0.004) were observed before and after admission, respectively. Temporal analysis revealed a stationary trend (p=0.41).

**Conclusion:**

In infants with acute bronchiolitis, younger age, presence of atelectasis, and higher Wood-Dowes-Ferres scores at admission were associated with invasive ventilation. Medications were frequently administered to the patients before and during hospitalization. This study emphasizes the need to consider these factors when caring for these infants and for clinical protocols to reduce the use of medications before and during hospitalization.

## In Brief

Infants with acute viral bronchiolitis admitted to a pediatric ward were observed between January 2020 and March 2023. Younger age, higher Wood-Downes-Ferres scores at admission, and the presence of atelectasis were associated with invasive ventilation. An increase in bronchodilator use and a decrease in corticosteroid use were observed before and after admission.

## Highlights

■ This single-center study evaluated the frequency and factors associated with invasive ventilation in infants with acute viral bronchiolitis on a pediatric ward.
■ The study revealed an invasive ventilation rate of 6%, associated with age, presence of atelectasis, and Wood-Downes-Ferres score at admission.
■ Many interventions with poor scientific evidence were used.
■ This study highlights the need to implement clinical protocols for the management of acute viral bronchiolitis, including all levels of care, as these infants are initially managed outside the hospital setting.

## Graphical abstract

**Figure:**
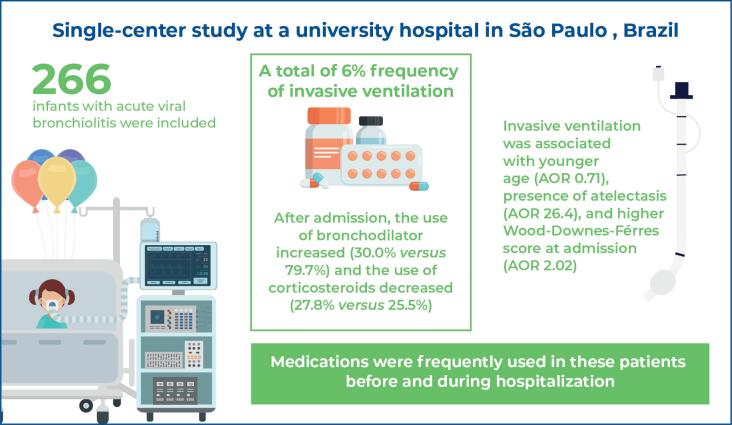

## INTRODUCTION

Acute viral bronchiolitis (AVB) is characterized by the non-specific inflammation of small-caliber airways in infants under 24 months, primarily caused by the respiratory syncytial virus (RSV), among others.^(1,2)^ Acute viral bronchiolitis is the leading cause of hospitalization for respiratory disease during infancy, with a rate of 57%.^(2,3)^

Acute viral bronchiolitis can be more severe in preterm infants, those with congenital heart or lung diseases, immunodeficiencies, neurological diseases, or anatomical defects of the respiratory tract.^(4)^ There are many available treatments, including corticosteroids and bronchodilators.^(1,5)^ Furthermore, the literature recommends respiratory support, supplemental oxygen, and airway clearance as supportive treatments.^(6,7)^

With the advent of the coronavirus disease (COVID-19) pandemic in 2020, caused by the SARS-CoV-2 virus, there has been a worldwide restriction on the movement of people, consequently limiting the spread of respiratory viruses.^(8)^ A study comparing hospitalizations for AVB at the beginning and end of the COVID-19 pandemic showed fewer hospitalizations during the 2020–2021 period, with an increase from 2022 to 2023, which caused health system overload, suggesting a change in seasonality patterns.^(9)^ The knowledge of factors associated with respiratory failure, especially in scenarios different from the intensive care unit, such as the pediatric ward, may help identify those who need specific care, improving their outcomes. Thus, contemporary studies on disease behavior, severity, and instituted treatments are appropriate.

## OBJECTIVE

To analyze the factors associated with invasive ventilation in infants hospitalized with acute bronchiolitis during the COVID-19 pandemic and to identify treatments instituted before and during hospitalization.

## METHODS

This single-center observational retrospective study was conducted at a university hospital in São Paulo, Brazil, and retrospectively covered the period from January 2020 to March 2023. The pediatric ward consisted of 12 beds, lacked a protocol for AVB management, and had no intensive care beds during the study period. The multidisciplinary team included pediatricians, nurses, nurse technicians, respiratory therapists, nutritionists, and occupational therapists. All patients with AVB received daily support from respiratory therapists.

The inclusion criteria were infants aged 0–24 months, diagnosed with AVB, and admitted to the pediatric ward. The exclusion criteria were the unavailability of patient information or a previous record or history of respiratory symptoms, which excluded the diagnosis of AVB.

The diagnosis of AVB was made on a clinical basis and defined as the first episode of upper respiratory tract (runny nose, sneezing) and lower respiratory tract (tachypnea, wheezing, coughing, crackling, and use of accessory muscles) symptoms in infants aged <2 years.^(10)^ The data were collected from electronic medical records and entered into the Research Electronic Data Capture (REDCap) questionnaire. All hospitalizations were tracked, with a focus on those aged <2 years with the 10^th^ International Classification of Diseases (ICD-10) diagnosis at admission codes J21 (acute bronchiolitis), J21.0 (acute bronchiolitis due to respiratory syncytial virus), or J21.8 (acute bronchiolitis due to other specified microorganisms). The infants’ characteristics, Wood-Downes-Ferres (WDF) score at admission, treatments instituted before and during hospitalization (nebulized hypertonic saline, bronchodilators administered by metered dose inhaler with spacer or intravenous, and corticosteroids via systemic route), viral panel results (rapid tests, performed by immunochromatography), presence of atelectasis, consolidation on chest radiography, and maximum respiratory support were investigated, as well as invasive ventilation, non-invasive ventilation, and nasal catheter use. High-flow nasal cannulas were not used during the study period. All abnormalities were detected by chest radiography.

This study was approved by the Research Ethics Committee of *Universidade Federal de São Carlos* (CAAE: 68765123.1.0000.5504; # 6.065.043). An exemption from the requirement for informed consent was obtained.

The collected data were analyzed using Stata software (version 18.0; Stata Corp, L.C.). Normality was assessed using the Shapiro–Wilk test. Medians and interquartile ranges (IQRs), frequencies, and 95% confidence intervals (95% CIs) were calculated. Differences between medians were analyzed using the Mann–Whitney test, and differences between proportions were analyzed using the chi-square or McNemar test. Cases that progressed to invasive ventilation were also analyzed. We compared infant characteristics, treatment institution, viral panel results, and chest radiographic abnormalities between the intubated and non-intubated groups. Variables with a p<0.20 in the bivariate analyses were tested in a stepwise multivariate logistic regression, considering the need for invasive ventilation (yes/no) as the outcome variable. Crude and adjusted odds ratios (ORs) and 95%CIs were calculated. Cases were investigated over time using Prais–Winsten regression, with time measured in months. A positive trend (p<0.05) was considered an increase, and a negative trend was considered a decrease. Trends were considered stationary at p>0.05. Statistical significance was set at p<0.05. This study adhered to the STROBE guidelines for observational studies.^(11)^

## Results

The study sample comprised 266 participants. Initially, 2,005 records were investigated; however, 1,739 were excluded because they were not the first respiratory episode or the clinical diagnosis was not AVB according to the medical records. The median age was 5 months (IQR=2–10), 120 (45.1%) were male, and the median weight at admission was 6.9 kg (IQR=4.8–9.0).

On admission, the median symptom duration was 3 days (IQR=2–6). Twenty infants (7.5%) had comorbidities, and premature birth was the most frequent comorbidity, occurring in 10 patients. Before admission, corticosteroids were used in 74 patients (27.8%), inhaled bronchodilators in 80 (30%), and antibiotics in 44 (16.5%). After admission, five infants used venous bronchodilators and 207 used inhaled bronchodilators. A comparison of treatments before admission and during hospitalization revealed an increase in the use of bronchodilators and a reduction in the use of corticosteroids (Table 1); however, 28 infants (37.8%) who used corticosteroids before admission continued receiving them, and 74 (92.5%) of those who used bronchodilators continued to use them.

**Table 1:** Treatments instituted before admission and during hospitalization

Treatment instituted	Before admission n (%)	During hospitalization n (%)	p value*
Nebulized hypertonic saline	-	148 (55.6)	-
Bronchodilator	80 (30.0)	212 (79.7)	<0.001
Corticosteroids	74 (27.8)	68 (25.5)	0.004

* p-value associated with the McNemar test.

The maximum respiratory support used during hospitalization was a nasal catheter in 158 (59.4%) patients, non-invasive ventilation in 33 (12.4%), and invasive ventilation in 16 (6%). The median length of hospital stay was 5 days (IQR=3–7), and those who used bronchodilators had a longer length of stay (p=0.01). The median WDF at admission was 3 (IQR=2–3).

Rapid tests for viral detection were performed; 86/186 (47.8%) patients were RSV-positive, 13/139 (9.3%) were positive for SARS-CoV-2, and 4/155 (2.5%) were positive for influenza virus. Sixteen infants (6%) were intubated, and no deaths were observed. Comparisons between those who were intubated and those who were not revealed differences in age, weight at admission, WDF at admission, presence of atelectasis or consolidation on chest radiographs, and length of hospital stay (Table 2).

**Table 2:** Characteristics, treatments instituted, viral positivity, and radiograph abnormalities between infants with acute viral bronchiolitis who were intubated and those who were not

Variable		n	Not intubated (n=250)	Intubated (n=16)	p value*
Infants’ characteristics and exams					
	Age in months, median (IQR)	266	5 (2–11)	2 (1-3)	0.0014
	Weight at admission in kg, median (IQR)	263	7.0 (5.0–9.1)	4.8 (4.1–5.4)	0.0006
	Male sex, n (%)	266	140 (56)	6 (37.5)	0.2149
	Comorbidity, n (%)	266	19 (7.6)	1 (6.2)	0.83
	Prematurity, n (%)		9 (3.6)	1 (6.2)	0.58
	Wood-Downes-Ferres score at admission, median (IQR)	266	3 (2–3)	3.5 (2–4.5)	0.0385
	Presence of atelectasis#, n (%)	197	32 (17.39)	8 (61.54)	<0.0001
	Presence of consolidation (s), n (%)	197	16 (8.7)	7 (53.8)	<0.001
	Length of stay in days, median (IQR)	266	5 (4–8)	1.5 (1–3)	<0.0001
Viral tests positivity£, n (%)					
	Respiratory Syncytial Virus	186	82 (46.8)	7 (63.6)	0.280
	SARS-CoV-2	139	13 (5.20)	0 (0)	-
	Influenza virus	155	4 (2.7)	0 (0)	-
Treatments instituted before admission, n (%)					
	Bronchodilator	266	75 (30)	5 (31.25)	0.933
	Corticosteroids	266	68 (27.2)	6 (37.5)	0.4899
Treatments during hospitalization, n (%)					
	Bronchodilator	266	194 (77.6)	13 (81.25)	0.8067
	Corticosteroids	266	65 (26)	3 (18.75)	0.6270
	Nebulized hypertonic saline	266	141 (56.4)	7 (43.75)	0.3964

* p-value associated with the Mann–Whitney or Chi-square test;

# percent calculated according to the number of infants who underwent chest radiograph, 13 in the intubated group and 184 in the not intubated group;

£ percent calculated according to the number of infants who performed tests, for respiratory syncytial virus, 11 in the intubated group and 175 in the not intubated group, for SARS-CoV-2, eight in the intubated group and 131 in the not intubated group, and for Influenza virus, 11 in the intubated group and 144 in the not intubated group.

The multivariate logistic regression model revealed that younger age (AOR=0.71), presence of atelectasis (AOR=26.4), and higher WDF at admission (AOR=2.02) were associated with invasive ventilation (Table 3).

**Table 3:** Factors associated with invasive ventilation: multivariate logistic regression analysis

Variable	Crude odds ratio	p value	Adjusted odds ratio	95%CI	p value*
Age	0.77	0.008	0.71	0.53–0.96	0.02
Wood-Downes-Ferres score at admission	1.65	0.005	2.02	1.03–3.95	0.03
Presence of atelectasis	7.6	0.0001	26.4	3.85–182.18	0.001

Model adjusted for male sex and length of stay.

* p-values associated with multivariate logistic regression.

**Figure 1:**
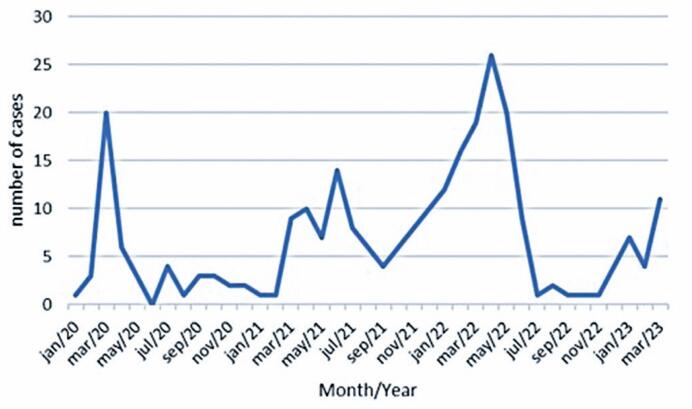
Acute viral bronchiolitis cases over time, from 2020 to 2023

A seasonal pattern of cases over time was observed without a trend (p=0.41) (Figure 1).

## Discussion

Our study revealed a 6% frequency of invasive ventilation in hospitalized infants with AVB. Specifically, younger age, atelectasis, and higher WDF at admission were associated with invasive ventilation. Medications were frequently used before and during hospitalization, with increased bronchodilator and decreased corticosteroid use after admission.

The frequency of invasive ventilation observed in the present study was within the range reported previously. This frequency varied in other studies, mostly according to the scenario studied, and was higher among patients in intensive care. As the institution did not have a pediatric intensive care unit during the study period, the admitted infants did not have an a *priori* indication for intensive care. Marlow et al. observed a 23.0% frequency of invasive ventilation in infants admitted to the intensive care unit.^(12)^ Camporesi et al. observed a 12.4% frequency of invasive ventilation in infants with AVB in the emergency departments of Latin America between March and December 2022.^(13)^ Between 2012 and 2014, an invasive ventilation frequency of 5.6% was registered in infants with AVB in the pediatric intensive care unit in Porto Alegre, Brazil.^(14)^ Conversely, Torres et al. observed a 3.2% frequency of invasive ventilation in a large database of infant hospitalizations in Miami, United States.^(15)^ We want to emphasize that the shorter length of stay for infants who were intubated occurred because, during the study period, there was no pediatric intensive care unit in the institution, and these patients were immediately transferred after intubation. The pediatric intensive care unit was inaugurated in April 2023.

In this study, age, WDF at admission, and the presence of atelectasis were associated with invasive ventilation. Camporesi et al. observed that age was associated with mechanical ventilation in the emergency department and that the probability of the need for mechanical ventilation decreased over time.^(13)^ Younger infants have different respiratory mechanics and immature immune systems, which may explain the poorer outcomes.^(16)^ In addition, the WDF is one of the most widely used scores for assessing the clinical severity of AVB and is an objective clinical tool that has already been used in Brazil in infants with AVB.^(17)^ Santos et al. conducted a clinical trial with infants with AVB who underwent either non-invasive ventilation or a high-flow nasal cannula and were considered mild or moderate cases, a scenario different from the present study. A WDF >8 is the criterion for failure, indicating the need for mechanical ventilation.^(18)^ The presence of atelectasis causes lung aeration impairment and has been associated with the need for invasive mechanical ventilation.^(19)^ In a case–control study conducted between 2014 and 2020, Shi et al. developed a prediction model for continuous positive airway pressure failure in infants with AVB. They observed that intubated infants had laboratory findings of atelectasis, lung consolidation, and other conditions. Atelectasis was associated with an elevated risk score.^(20)^ This study, which aligned with the literature, reinforces the importance of avoiding atelectasis in infants with AVB and the immediate treatment of the condition when present, especially considering that younger infants are affected differently. Furthermore, it indicates the need for the systematic application of an objective score to evaluate these infants and detect those at risk for invasive ventilation as soon as possible. It is important to emphasize that, in the present study, all infants received respiratory therapy independent of atelectasis, which does not seem to have influenced the results.

We observed that some pharmacological interventions were initiated before admission and then continued or discontinued during hospitalization. Other interventions were initiated during hospitalization. When an infant was admitted for bronchodilator use, the medication was not discontinued in 92.5% of cases. The use of corticosteroids before and during hospitalization decreased. However, the use of bronchodilators before and during hospitalization has increased in recent years. AVB management varies and must be based on clinical protocols.^(21)^ In a systematic review that included 32 AVB guidelines, 22 guidelines did not recommend bronchodilator use, three recommended it, and no guideline recommended corticosteroid use. Nebulized hypertonic saline was recommended in eight guidelines, while seven did not, and the authors emphasized that many guidelines were built with methodological concerns.^(22)^ Pittet et al. observed AVB management in the emergency department in Geneva during different periods and reinforced the need for studies investigating the use of unnecessary interventions. The institution does not recommend their use; however, the use of bronchodilators varied between 17.3% and 23.7%, and that of corticosteroids between 0.5% and 2.2%.^(23)^ Curiously, the authors discuss several barriers that need to be overcome, such as parents’ pressure to "do more" and physicians’ apprehension of relying only on clinical assessment without investigations. Implementing the AVB protocol and educating health professionals in primary care and emergency departments is important, as patients are initially evaluated and managed in these scenarios.^(24)^ These pharmacological interventions that lack scientific evidence of their efficacy are frequent.^(25)^ A systematic review reinforced that the therapeutic approach recommended for AVB remains unchanged and limited to respiratory and metabolic support, and there is a challenge to implement and improve adherence to AVB protocols.^(23,26)^ It is important to note that the institutions studied did not have an AVB protocol during the study period, and the observed treatments reflected the health professionals’ knowledge and clinical practices.

Our study has some limitations. First, because this was an observational retrospective study, we assessed the available information and did not infer any causality. Second, the institution did not have a pediatric intensive care unit during the study, and the intubated patients were transferred as soon as possible. Finally, the small number of intubated infants may have limited the statistical relevance of risk factors for invasive ventilation.

## CONCLUSION

The frequency of invasive ventilation was 6% in infants with acute bronchiolitis. Younger age, the presence of atelectasis, and a higher Wood-Downes-Ferres score at admission were associated with invasive ventilation. Medications were frequently administered to the patients before and during hospitalization. This study emphasizes the need to consider these factors when caring for these infants and highlights the demand for clinical protocols to reduce the use of medications before and during hospitalization.
